# Alzheimer's disease and Type 2 diabetes mellitus: the cholinesterase connection?

**DOI:** 10.1186/1476-511X-5-28

**Published:** 2006-11-11

**Authors:** Appa Rao Allam, Gumpeny Ramachandra Sridhar, Hanuman Thota, Changalasetty Suresh Babu, Akula Siva Prasad, Ch Divakar

**Affiliations:** 1 Department of Computer Sciences and Systems Engineering, Andhra University, Visakhapatnam 530 003, India; 2 Endocrine and Diabetes Centre, 15-12-16 Krishnanagar, Visakhapatnam 530 002, India; 3 Department of Computer Sciences, Acharya Nagarjuna University, Guntur, India; 4 Department of Computer Science, Gandhi Institute of Technology And Management, Visakhapatnam, India

## Abstract

Alzheimer's disease and type 2 diabetes mellitus tend to occur together. We sought to identify protein(s) common to both conditions that could suggest a possible unifying pathogenic role. Using human neuronal butyrylcholinesterase (AAH08396.1) as the reference protein we used BLAST Tool for protein to protein comparison in humans. We found three groups of sequences among a series of 12, with an E-value between 0–12, common to both Alzheimer's disease and diabetes: butyrylcholinesterase precursor K allele (NP_000046.1), acetylcholinesterase isoform E4-E6 precursor (NP_000656.1), and apoptosis-related acetylcholinesterase (1B41|A). Butyrylcholinesterase and acetylcholinesterase related proteins were found common to both Alzheimer's disease and diabetes; they may play an etiological role via influencing insulin resistance and lipid metabolism.

## Background

Alzheimer's disease and type 2 diabetes mellitus occur with increasing frequency as age advances. Besides, the development of one increases the risk of the other [1]. Epidemiological studies have shown an association of diabetes mellitus and Alzheimer's disease. A population-based historical cohort study estimated that the risk of Alzheimer's disease increased with adult onset diabetes mellitus [2] A longitudinal study of 1,262 elderly subjects without dementia at baseline, adjusted relative risk of Alzheimer's disease among persons with diabetes was 1.3 [95% CI: 0.8, 1.9], [3]. In a more recent community-based study among 1301 dementia-free persons aged 75 and above, diabetes mellitus was associated with subsequent development of Alzheimer's disease [4]. Similarly patients with Alzheimer's disease were more vulnerable to developing impaired fasting glucose and type 2 diabetes mellitus [5]. A variety of mechanisms has been postulated in the risk of Alzheimer's disease and type 2 diabetes mellitus: metabolic abnormalities of insulin resistance (dyslipidemia, hypertension), hyperglycemia per se or insulin, by disturbing synaptic plasticity, learning and memory [6].

The enzyme butyrylcholinesterase (EC 3.1.1.1.8) does not have a well-defined physiological function, although it may modulate the phenotypic expression of dyslipidemia and insulin resistance. It is affected by dietary factors, obesity, and dyslipidemia [5,7,8]

While acetylcholinesterase in the brain is chiefly localized to neurons, butyrylcholinesterase is primarily associated with glial cells and endothelial cells [9].

Butyrylcholinesterase was studied in relation to both type 2 diabetes mellitus and Alzheimer's disease in different ethnic groups [10-13].

Despite originating from the endoderm, the pancreas is highly innervated and shares molecular similarities with brain at the level of transcriptome and proteome [14]. Localized and progressive amyloidosis is characteristic of both type 2 diabetes and Alzheimer's disease. Neurofibrillary tangles, the manifestations of pancreatic islet neurodegeneration have not been as extensively studied in diabetes mellitus as in Alzheimer's disease. Cytotoxic effects of fibril in both conditions involve an interaction of cell membranes with mis-folded insoluble peptides [15]. The two may therefore be mediated by common regulatory elements.

Considering that butyrylcholinesterase was associated with both type 2 diabetes mellitus and with Alzheimer's disease, and that leads existed for the a role of butyrylcholinesterase in their pathogeneses, we chose it as the reference protein. In addition, genetic variants of the enzyme exist, which may play a role in biological fitness of individuals [16]. Identification of such sequences would provide leads for further understanding etiological, therapeutic or prognostic aspects [17-20].

Data was retrieved from National Centre for Biotechnology Information (NCBI). We took the amino acid sequence of human neuronal butyrylcholinesterase (AAH08396.1) as the reference protein and copied it to Word programme in FASTA format. We used BLAST tool in NCBI, and performed protein to protein comparison in humans from the available databases. Taking E-value between 0–12, we obtained 12 sequences that were common to both Alzheimer's disease and diabetes mellitus (Table 1). The E-value refers to the probability due to chance, that there is another alignment with a similarity greater than the given similarity score. In general, An E<e^-5 ^of an alignment means that that alignment is highly unique, and not due to error, whereas an E ≥ e^-6 ^means that the alignment might be strong, but more research is needed to verify. The lower the E value, the more significant the score.

**Table 1:** Sequences common to Alzheimer's Disease and Diabetes mellitus

SeqA Name	Len(aa)	SeqB Name	Len(aa)	E-Score*
1 AAH08396.1	64	CHLE_HUMAN	602	4e-34
2 AAH08396.1	64	NP_000046.1	602	4e-34
3 AAH08396.1	64	NP_000656.1	614	1e-16
4 AAH08396.1	64	BAD97163.1	614	1e-16
5 AAH08396.1	64	1F8U|A	583	2e-16
6 AAH08396.1	64	AAO32948.1	526	3e-16
7 AAH08396.1	64	AAH94752.1	640	2e-15
8 AAH08396.1	64	AAK21003.1	94	6e-13
9 AAH08396.1	64	1P0Q|A	529	3e-05
10 AAH08396.1	64	1VZJ|H	40	0.001
11 AAH08396.1	64	AAU43801.1	617	0.14
12 AAH08396.1	64	1B41|A	539	0.20

* The E-value refers to the probability due to chance, that there is another alignment with a similarity greater than the given similarity score

The following sequences were obtained (Table 1):

Essentially the sequences may be grouped into butyrylcholinesterase precursor K allele (NP_000046.1), acetylcholinesterase isoform E4-E6 precursor (NP_000656.1), and apoptosis-related acetylcholinesterase (1B41|A).

Dementia and diabetes increase with age. This is related to the occurrence of atherosclerosis, advanced glycation end product, oxidative stress and the deposit of amyloid. [1,5]. Neurofibrillary tangles are deposited in brain of Alzheimer's disease, along with senile plaques, which also contain extraceullular deposits of human amyloid beta peptide. Amyloid deposits, consisting of elongated unbranched fibrils that bind to Congo Red dye, consist of among others, acetylcholinesterase and apolipoprotein E [21]. Amyloid fibrils are also seen in pancreatic beta cells in type 2 diabetes mellitus [22]. Association of butyrylcholinesterase with Alzheimer's disease showed conflicting results: some showed positive associations of BChE polymorphism acting in synergy with APOE 4 [10], others no significant differences while yet others protection among women. The rationale for looking at the association is that BChE gene codes for butyrylcholinesterase, which is found in amyloid plaque [23]. BChE acts in concert with apolipoprotein E which are both astrocyte proteins that interact with lipoproteins after being released into the circulation by the liver.

The variable association of BchE and variants with Alzheimer's disease [10,23-25] was attributed to ethnic differences in frequency of BChE variants, perhaps due to hidden population admixture, or of variant BChE being in 'linkage disequilibrium with an as yet unidentified AD susceptibility gene in some populations [12].

Studies have linked BchE with the pathogenesis of Alzheimer's disease and diabetes mellitus. BchE may modify the risk of Alzheimer's disease either alone, or in synergy with apoE-epsilon 4 [13]. Its active involvement is also suggested by plaque area of demented brains having higher BchE, and by its localization in neurofibrillary tangles, a pathological hallmark of Alzheimer's disease. A recent study showed that BchE attenuates amyloid formation by an interaction of C terminus with soluble species of beta amyloid in a polar environment [26]. It may prolong nucleation phase and reduce propogation phase of fibril formation and suppress amyloid fibril formation enhanced by purified AchE-S [26]. Similarly selective inhibition of BchE in aged rats improved cognitive navigation [9]. In cultured neuroblastoma cells, selective BchE inhibition reduced beta amyloid precursor protein and beta amyloid peptide levels. It is supportive in the hydrolysis of acetylcholinesterase and can partly compensate for the action of AchE. While AchE levels are reduced early in Alzheimer's disease, BchE levels rise with disease; selective BchE inhibition may be useful to ameliorate cholinergic defect. Therefore BchE may have a role in coregulating local concentrations of acetylcholinesterase in Alzheimer's disease.

BchE may be involved in the pathogenesis of type 2 diabetes either by way of amyloid fibrils or by modifying other risk factors of insulin resistance. Amyloid fibrils in pancreatic islets produce excessive superoxide radicals, lipid peroxidation and nitric oxide inactivation, contributing to apoptosis of beta cells [15]. K variant of BchE have propensity for beta sheet formation, which may be related to amyloidogenesis. In addition, a locus on chromosome 3q27, which is close to the chromosomal locus of BchE was linked to type 2 diabetes mellitus in a French population [27].

In addition, increased BchE may predispose aging cells to oxidative stress [16].

Conventional association studies with evaluation of limited phenotypes are laborious, and do not always give unambiguous answers proportionate to the effort incurred [15]. With the availability of nucleotide and amino acid sequences, we performed a bioinformatics approach to study butyrylcholinesterase-related proteins in Alzheimer's disease and in type 2 diabetes mellitus.

Comparative genomic study across species provides a more broad-based picture of proteins in terms of evolution; earlier we showed that butyrylcholinesterase (EC 3.1.1.1.8) and its variants were spread in mammals consistent with their evolutionary space, and that homologous sequences were expressed in other life forms, including plants and bacteria [11,28].

*Butyrylcholinesterase *(NP_000046.1, Table 1) whose gene is localized to a single autosomal location at 3q26 has extensive homology to other cholinesterases of various species. More than 30 genetic variants of BchE have been reported, and the number is increasing. The occurrence of such preadaptative pharmacogenetic variants was attributed to either balanced polymorphisms or to neutral mutations [29].

A recent *in vitro *study showed that BchE attenuates amyloid fibril formation [9]. The association of BchE K variant in both Alzheimer's disease [10] and type 2 diabetes mellitus suggests a possible pathogenic role in both diseases.

*Acetylcholinesterase *(NP_000656.1, Table 1) regulates cholinergic nerve and neuromuscular transmission; the gene encoding this protein is mapped to chromosome 7. Acetylcholinesterase belongs to the family of hydrolases along with butyrylcholinesterase, with which it has 53% sequence homology [17].

Acetylcholinesterase-like proteins were shown to mediate cytoarchitectural changes that support neurotogenesis [30]. It also has a role in human neocortical neuroplastic processes [31].

There is substantial structural and functional evidence linking acetylcholinesterase to Alzheimer's disease: a region near C-terminus is weakly homologous to the N-terminus of amyloid-beta peptide [32]. In a comparative study on neurotoxicity comparison, human amyloid-beta peptide fibrils complexed with acetylcholinesterase showed greater toxicity than amyloid beta peptide fibrils along [21]. Acetylcholinesterase may potentiate both amyloid deposition and the toxicity of such deposits.

A recent study showed that hydrogen peroxide may be a regulator of AchE [33]. The reaction is similar to that which occurs in vitiligous skin, resulting in severe oxidative stress [34]. Similarly, increased membrane AchE activity was attributed to high homocysteine levels, which are correlated with neurological problems [34]. Oxidative stress could result from hyperhomocystinemia, leading to neuronal apoptosis [35] and to insulin resistance [36]. Similar suppression of butyrylcholinesterase has been shown to occur with homocysteine, probably acting via free radicals [35]

*Apoptosis-related acetylcholinesterase *(AAO32948.1) is an alternatively-spliced variant expressed in blood cells, associated with apoptosis. It could be a potential marker and a regulator of apoptosis, being expressed in apoptotic cells. Similarly it could be modulate pro-inflammatory cytokines in macrophages in the periphery, and be differentially expressed by stress in the periphery and in the central nervous system [37].

Insulin signaling abnormalities could be the underlying mechanism affecting the outcome of Alzheimer's disease; insulin resistance and disordered degradation of amyloid seem to link diabetes mellitus with Alzheimer's disease [38]. Insulin dysregulation could act in a variety of ways including decreased cortical glucose utilization, oxidative stress formation of advanced glycated proteins, increased neurofibrillary formation and increased b-amyloid aggregation through inhibition of insulin-degrading enzyme [39]. Insulin resistance could therefore be a link between Alzheimer's disease and type 2 diabetes mellitus [40]. Butyrylcholinesterase may be indirectly involved in the pathogenesis of insulin resistance [41]. It has been hypothesized that peripheral insulin resistance can affect CNS insulin levels, cognition and amyloid beta levels [40]. Peripheral insulin resistance downregulate insulin uptake at the blood brain barrier and lead to CNS insulinopenia. Since insulin promotes intracellular amyloid beta release and alters expression of insulin degrading enzyme, low brain insulin levels can lead to amyloid beta accumulation in neurons. Peripheral insulin resistance may also inhibit clearance of amyloid beta from the brain to the periphery, either by blocking its transport from the brain or by interference with clearance in peripheral sites. Thus there could be a combination of accumulation of amyloid beta, with decreased clearance, both due to insulin resistance [40].

Antidiabetic drugs could be potentially useful in treating Alzheimer's disease: PPAR gamma agonists, by improving insulin sensitivity, decreasing inflammation and improving cerebral energy metabolism; intranasal insulin, by restoring brain insulin levels in Alzheimer's disease [40].

Another intriguing aspect is the occurrence of variant butyrylcholinesterases with low enzyme activity in certain ethnic populations [16]. It would be instructive to study the prevalence and course of both insulin resistance and Alzheimer's disease in these individuals who may be considered to be on butyrylcholinesterase inhibitors. Such studies are particularly instructive, considering that other forms of treatment such as beta-secretase inhibitors may be associated with potential negative consequences [42]. Individuals with BchE deficiency have been known to survive without any apparent adverse physiological consequences.

In summary, esterase group of enzymes may be an underlying thread in the coexistence of Alzheimer's disease and diabetes mellitus.

At present however, one cannot impute a direct cause-effect relationship among the variables discussed here (Alzheimer's disease, type 2 diabetes mellitus, insulin resistance, butyrylcholinesterase), although there are epidemiological, biochemical, pathological and now computational biological leads pointing to an association. In line with emerging paradigms of *"in vivo *to *in silico *biology and back" [43], our results offer direction in the iterative processes that drive biology forward in comprehending biological phenomena.

## Competing interests

The author(s) declare that they have no competing interests.

## Authors' contributions

*GRS *participated in the design of the study, interpretation of the results and prepared the manuscript. *TH, SB, SP and DC *participated in the design of the study, performed the bioinformatics aspects and participated in the preparation of the manuscript. *AAR *participated in the design of the study, guided in the bioinformatics aspects, and participated in the interpretation of the results and in the preparation of the manuscript. All authors read and approved the final manuscript.
